# Characteristics of neonatal hypoxic-ischemic encephalopathy at high altitude and early results of therapeutic hypothermia

**DOI:** 10.1186/s12887-023-04421-3

**Published:** 2023-12-01

**Authors:** Jia Li, Guofei Zhang, Xiaorong Wang, Cuozhen Qiangba, Xiaoyan Song, Rouyi Lin, Chantao Huang, Xiaoying Yang, Shuyao Ning, Jian Zhang, Haiyan Liao, Siyuan Xie, Zhen Suo, Haiying Qi, Zhen Yu, Runling Shi, Yanli Yao

**Affiliations:** 1 Clinical Physiology Laboratory, Institute of Pediatrics, Guangzhou Women and Children’s Medical Center, Guangzhou Medical University, Guangdong, China; 2 Clinical Physiology Laboratory, Capital Institute of Pediatrics, Beijing, China; 3 NICU, Qinghai Red Cross Hospital, Xining, Qinghai China; 4 NICU, Qinghai Women’s and Children’s Hospital, Xining, Qinghai China; 5 NICU, Lhasa People’s Hospital, Lhasa, Tibet, China; 6 NICU, Nanfang Hospital, Guangzhou, Guangdong China; 7 Department of Radiology, Nanfang Hospital, Guangzhou, Guangdong China; 8 Department of Electroneurophysiology, Guangzhou Women and Children’s Medical Center, Guangzhou Medical University, Guangdong, China; 9 Department of Echocardiography, Lhasa People’s Hospital, Lhasa, Tibet, China; 10 Department of Echocardiography, Qinghai Women’s and Children’ Hospital, Xining, Qinghai China

**Keywords:** Neonatal hypoxic ischemic encephalopathy, Therapeutic hypothermia, High altitude, Electroencephalogram, Magnetic Resonance Imaging

## Abstract

**Background:**

Altitude hypoxia and limited socioeconomic conditions may result in distinctive features of neonatal hypoxic-ischemic encephalopathy (HIE). Therapeutic hypothermia (TH) has not been used at altitude. We examined characteristics of HIE and early outcomes of TH in 3 centers at two high altitudes, 2 at 2,261 m and 1 at 3,650 m.

**Methods:**

The incidence of HIE at NICUs was noted. TH was conducted when personnel and devices were available in 2019~2020. Standard inclusion criteria were used, with the addition of admission age >6 hours and mild HIE. Demographic and clinical data included gestational age, gender, weight, Apgar score, ethnics, age on admission, age at TH and clinical degree of HIE. EEG was monitored for 96 hours during hypothermia and rewarming. MRI was performed before discharge.

**Results:**

There was significant difference in ethnics, HIE degree, age at TH across 3 centers. The overall NICU incidence of HIE was 4.0%. Among 566 HIE patients, 114 (20.1%) received TH. 63 (55.3%) patients had moderate/severe HIE. Age at TH >6 hours occurred in 34 (29.8%) patients. EEG discharges showed seizures in 7~11% of patients, whereas spikes/sharp waves in 94~100%, delta brushes in 50~100%. After TH, MRI showed moderate to severe brain injury in 77% of patients, and correlated with center, demographic and clinical variables (Ps≤0.0003). Mortality was 5% during hospitalization and 11% after discharge until 1 year.

**Conclusions:**

At altitude, the incidence of HIE was high and brain injury was severe. TH was limited and often late >6 hours. EEG showed distinct patterns attributable to altitude hypoxia. TH was relatively safe.

**Trial registration:**

The study was registered on February 23, 2019 in Chinese Clinical Trial Register (ChiCTR1900021481).

**Supplementary Information:**

The online version contains supplementary material available at 10.1186/s12887-023-04421-3.

## Introduction

Neonatal hypoxic ischemic encephalopathy (HIE) remains a major cause of death, acute brain injury and neurodevelopmental impairment [1]. Therapeutic hypothermia (TH) induced within 6 hours after birth in moderate to severe HIE reduced mortality and neurodevelopmental impairment in the initial trials [2–4]. Later, broader criteria have been evolved to include mild HIE [5–7] and admission > 6 hours [8]. TH is the standard of care in developed countries [7, 9]. but not so in developing countries where 99% of the disease burden occurs [10], and where most of the high-altitude regions are located [11]. A study from Lhasa (altitude 3,650 m) reported a NICU incidence of 20% [12]. There has been no other report about clinical characteristics of HIE and TH application at high altitudes.

HIE may have distinctive features at altitude to due hypoxia and socioeconomic limitations. Altitude hypoxia is related to placental abnormalities and intrauterine growth retardation [13, 14], which may compound cerebral hypoxic-ischemia insult at birth. Many mothers do not have regular pregnancy checks and give birth to babies in local communities and need to be transported to the centers in provincial capitals where the medical resources remain limited. Initiating TH before 6 hours can be difficult.

We examined clinical characteristics of HIE and early outcomes of TH conducted in 3 centers at two high altitudes, 2 in Xining (altitude 2,261 m) and 1 in Lhasa, Tibet (average 4,000 m).

## Patients and methods

TH was conducted at the NICUs of Qinghai Red Cross Hospital (Center 1) and Qinghai Women's and Children's Hospital (Center 2) in Xining and the Lhasa People’s Hospital in Lhasa (Center 3) in 2019~2020. The centers were classified according to the severity of electroencephalography (EEG) background abnormalities (see below).

No randomization was made given the confirmed effects of TH [2–4]. Eligibility criteria were identical to the published studies [2–4], except for mild HIE [5, 7], admission age > 6 hours [8], and simple congenital heart defects since their incidence in newborns is about 20 times higher compared to low altitude [15].

The NICU incidence of HIE retrospectively was reviewed in each center and calculated as the number of HIE patients divided by the total number of admissions to each NICU during the study period.

The study was approved by Qinghai Red Cross Hospital (No.0208), Qinghai Women's and Children's Hospital (No.2018023), and Xining and the Lhasa People’s Hospital in Lhasa (No.201811) institutional Research Ethics Board. Written and informed consent was obtained from the participants and parents of the participants involved in the study. All ethical principles relating to the Declaration of Helsinki and the Convention on Human Rights and Biomedicine were followed.

### Clinical management

#### Routine management

A uniform clinical management protocol was applied [16]. Administration of antiepileptic drugs included phenobarbitone as first-line medication, followed by midazolam as second-line medication.

#### Temperature management

A standard procedure of whole-body mild hypothermia [3, 4] was conducted using Blanketrol® III Hyper-Hypothermia System (Gentherm Medical, Cincinnati, OH, USA) [8]. The rectal temperature was maintained at 33~34 °C for 72 hours and then rewarmed at < 0.5 ºC/h until 36.5-37.5 ºC.

### Methods of measurements

#### Patient monitoring

All patients had monitoring of heart rate, blood pressure, respiratory rate, rectal temperature, and peripheral temperature at the great toe (MP30, Royal Dutch Philips Electronics Ltd., Amsterdam, Holland). Pulse oximetry was used to monitor SpO_2_.

#### *Cerebral oxygen saturation (ScO*_*2*_*)*

ScO_2_ was continuously measured using near-infrared spectroscopy (INVOS 5100C, Medtronic & Covidien, Troy, MI, USA) [17].

#### EEG monitoring

**Fig. 1:**
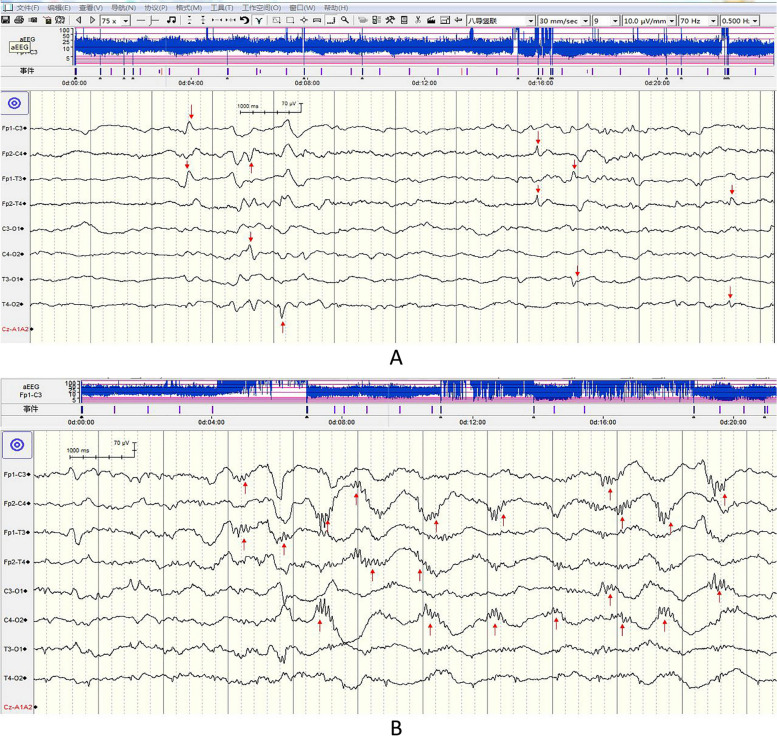
**A** Sharp waves (indicated by the red arrows) observed in a patient recorded at 6 hours of therapeutic hypothermia. **B** Delta brushes (indicated by the red arrows) observed in another

A Nicolet monitor (CareFusion, Middleton, Wisconsin, USA) was used with the international 10-20 system of electrode placement modified for neonates [18]. Seizures were defined as localized rhythmic paroxysmal activity>10 s [19]. The duration of seizures is the sum of seizure onsets. Spikes/sharp waves were defined as high amplitude (≥2.5 times of background voltage) and short duration (<200 ms) (Fig. 1A) [20]. Delta brushes consisted of slow waves (0.3e1.5 Hz) and superimposed fast activity (8e22 Hz) (Fig. 1B) [21]. EEG background was classified into 1 of 4 patterns based on a standard method [19].

#### *Brain* Magnetic resonance imaging *(MRI)*

MRI performed at 3T consisted of harmonized protocols and sequences including T1 and T2 weighted sequences, and diffusion-weighted imaging. The degree of brain injury was analyzed using the National Institute of Child Health and Human Development Neonatal Research Network (NICHD-NRN) classification into 0, 1A, 1B, 2A, 2B, or 3 according to the involvement of basal ganglia or thalamus, anterior or posterior limb of the internal capsule and other cerebral lesions [22].

#### Demographic and clinical data

Demographic data included gestational age, birthweight, gender, 5- and 10-minute Apgar scores, ethnics, born within the hospital (inborn) or transferred from communities (outborn), age on admission, and initiation age of TH. Clinical data included umbilical-cord venous blood /arterial blood gas if available and degree of HIE. During TH and rewarming period, patients were monitored for cardiac arrhythmia, persistent acidosis, skin changes (subcutaneous fat necrosis, etc), bloodstream infection, and major-vessel thrombosis. Clinical seizures were not recorded [23].

#### Study protocol

Consecutive patients were enrolled when the devices (one set was provided to each center) and personnel were available and consent was obtained. Upon initiation of the study, EEG and ScO were monitored for 96 hours including 72 hours of hypothermia and 24 hours of rewarming. Brain MRI was performed on day 5~22 (median 8) after birth. Clinical monitoring measures and ScO_2_ were collected in 4-hour periods. EEG was analyzed in 4-hour periods by a trained technologist (R.L) and brain MRI by a radiologist (C.H). Phone call follow-up was made to check the death rate until 1 year after discharge.

#### Statistical analysis

Data were described as mean±SD, median (range), or frequency (%) when appropriate. t-test, Mann-Whitney U test, or Chi-squared test was used when appropriate to examine the difference between 2 groups; ANOVA, Kruskal-Wallis test, or Chi-squared test was used when appropriate among 3 groups. The ethnics were classified according to historic order of migration, and centers according to the degree of EEG background abnormalities [24]. These variables were numbered 0, 1, or/and 2 from low to high respectively. Mixed linear regression for repeated measures was used to 1) analyze the temporal trend of variables (P_time_); 2) compare the difference in levels between groups. The parameter estimate and P value of the group/center effect (P_group_ or P_center_) indicate the difference in the level of each measure between groups/centers; 3) analyze correlations between measures. Polynomial transformation of time was tested regarding the best fit for time, in which the parameter estimate and P value of time (parameter estimate_time_ and P_time_) indicated earlier time, and those for time^2^ (parameter estimate_time_^2^ and P_time_^2^) indicated later time. All analyses were performed with SAS 9.4(SAS Institute Inc, Cary, NC). *P*<0.05 indicated significance.

## Results

### Demographic and clinical data

As shown in Table 1, there was significant difference across 3 centers in demographic and clinical data (Ps<0.05). Among 566 HIE patients, consent was obtained in 114 (20.1%) when the devices were available. Among 114 patients, 8 (7.0%) patients withdraw from TH due to financial reasons. There were no complications related to TH. 5 (4.8%) patients undergoing TH died of pneumonia, sepsis and respiratory failure on day 7~10 after birth in Center 1 and 2, and none in Center 3. 10 (out of 89, 11.2%) patients died after hospital discharge before 1 year old.

**Table 1:** Demographic and clinical data in the multicenter Study

**Measure**	**Center 1 (*n*=72)**	**Center 2 (*n*=20)**	**Center 3 (*n*=12)**	***P* value**
**Incidence of HIE in NICU**	399 (4.0%)	99 (2.5%)^b^	68 (6.1%)^a^	<0.001
**Patients undergoing hypothermia,** no.(%)	76 (19.0%)	25(25.2%)	13(19.1)	0.35
**Patients completing hypothermia,** no.(%)	72 (18.0%)	20 (20.2%)	12 (17.6%)	0.61
**Sex**				0.06
Male, n (%)	33 (45.8%)	15 (75%)	10 (83.3%)	
Female, n (%)	39 (54.2%)	5 (25%)	2 (16.7%)	
**Ethnic**				<0.001
Han, n (%)	50 (69.4%) ^a^	4 (20%) ^b^	0 (0%) ^b^	
Hui etc, n (%)	8 (11.1%)	9 (45%) ^a^	0 (0%)	
Tibetan, n (%)	14 (19.4%) ^b^	7 (35%)	12 (100%) ^a^	
**Gestational age** (Week)	39.76±1.84	39.65±1.32	39.17±1.53	0.69
**Birthweight (g)**	3158±514	3124±313	3180±605	0.22
**5-min Apgar score**	8 (0-10) ^d^	4 (0-9) ^c^	6 (3-9)	<0.001
**10-min Apgar score**	8 (0-10) ^d^	6 (0-10) ^c^	8 (5-10)	0.02
**Cord blood/first arterial blood pH**	7.29 (6.95-7.43)	7.27(6.87-7.43)	7.27(7.17-7.37)	0.29
**Base deficit (mmol/L)**	-8.3 (-25.3--3)	-12.7 (-29.3--3.5)	-12.0 (-23--4)	0.09
**Inborn:outborn,** n:n	48:24 ^a^	3:17 ^b^	6:6	<0.001
**Age at admission** (Hour)	1 (0-12)^d^	3 (1-17)^c^	1 (1-3)	<0.001
**Admission > 6 hours** n (%)	1 (1.4%)	3 (15%)^a^	0 (0%)	0.01
**Age at hypothermia** (Hour)	2 (0-12) ^c^	9 (3-20) ^d^	22 (4-45) ^d^	<0.001
**Starting hypothermia > 6 hours,** no.(%)	11 (15.3%) ^b^	15 (75%) ^a^	8 (67%) ^a^	<0.001
**Patients received mechanical ventilation** no.(%)	24 (33.3%)	11 (55%)	4 (33%)	0.12
**Clinical degree of HIE**				<0.001
Mild, n (%)	35 (48.6%) ^a^	3 (15.8%)	2 (16.7%)	
Moderate, n (%)	29 (40.3%)	6 (31.6%)	8 (66.7%)	
Severe, n (%)	8 (11.1%) ^b^	10 (52.6%) ^a^	2 (16.7%)	
**EEG abnormal discharges**	(*n*=68)	(*n*=18)	(*n*=10)	
Seizures, n (%)	5 (7.3%)	2 (11.1%)	1 (10.0%)	0.74
Spikes/sharp waves, n (%)	67 (98.5%)	17 (94.4%)	10 (100%)	0.36
Delta brushes, n (%)	35 (51.5%)	11 (61.1%)	10 (100%) ^a^	0.006
**Brain injury on MRI by NICHD-NRN**	(*n*=62)	(*n*=18)	(*n*=11)	<0.001
Normal/Mild, n (%)	11 (17.7%)^a^	0 (0.0%)	0 (0.0%)	
1B	7 (11.3%)	2 (11.1%)	1 (9.1%)	
2A	9 (14.5%)	2 (11.1%)	1 (9.1%)	
2B	34 (54.8%)	12 (66.7%)	7 (63.6%)	
3	1 (1.6%)	2 (11.1%)	2 (18.2%)	
**Hypothermia withdrawn,** n (%)	2 (2.6%) ^b^	5 (20%)^a^	1 (7.8%)	0.004
**Hospitalization time,** day (SD)	12.3 (8.4)	10.1(4.3)	11.1 (5.5)	0.56
**Death during hospitalization**	3 (3.9%)	2 (8.0%)	0(0%)	0.39
**Death before 1 year old,** n (%)	(*n*=60)	(*n*=17)	(*n*=12)	
	3 (5.0%)	3 (17.6%)	2 (16.7%)	0.12

*Abbreviation*: *HIE* Hypoxic-ischemic encephalopathy, *MRI* Magnetic resonance imaging, *NICHD-NRN* National Institute of Child Health and Human Development Neonatal Research Network classification

^a^higher than the expected value in the Chi-Square test, *P*≤0.001

^b^lower than the expected value in the Chi-Square test, *P*≤0.001

^c^significantly different from

^d^In Dunn's nonparametric comparison for post hoc test, *P*<0.05

### EEG and ScO_2_

EEG could be interpreted in 68 patients (mean duration 70.5±27.1 hours) in Center 1, 18 (65.0±30.4 hours) in Center 2, and 10 (80.0±29.8 hours) in Center 3. Detailed EEG features and statistical results of the changes over 96-hour monitoring and difference across 3 centers are shown in Additional file 1. EEG background abnormalities were significantly more severe over time and across 3 centers (P_time_ and P_center_<0.0001). Seizures occurred in 7~11% of patients in 3 centers. The duration of seizures decreased significantly over time (P_time_=0.002), without significant difference across 3 centers (P_center_=0.59). Spikes/sharp waves (Fig. 1A) occurred in 94.3~100% across 3 centers. The number of spike/sharp waves was related to time in a polynomial function, with an early decrease (P_time_=0.001) within 60 hours, followed by an increase (P_time_^2^=0.004). It significantly increased across 3 centers (P_center_=0.0004). Delta brushes (Fig. 1B) occurred in 50~100% across 3 centers, being the highest in Center 3 (*P*=0.006). The number of delta brushes trended to increase over time (P_time_<0.0001) without significant difference across centers (P_center_=0.65). ScO_2_ was related to time in a polynomial function, with an early increase peaking at about the end of TH, followed by a decrease (P_s_<0.0001). It increased significantly across 3 centers (P_center_.=0.01). After being adjusted by time and center, none of the EEG variables significantly correlated with demographic or clinical variables (Ps>0.40).

#### Brain MRI

MRI was performed on 91 patients (Table 1). 71.0~90.9% of patients in 3 centers had moderate or severe brain injuries (i.e., ≥2A in). In univariable regression, the degree of brain injury on MRI positively correlated with center, sex, ethnicity, HIE, initiation age of TH and negatively with birth weight (Ps<0.0001). In multivariable regression, the correlations with the above variables remained significant (Ps≤0.0015) (Table 2). The degree of MRI brain injury also significantly and positively correlated with EEG background abnormalities and the number of spikes/sharp waves (Parameter estimate=0.469, 0.0033 respectively, Ps<0.0001), but not seizures or the number of delta brushes (Ps>0.40).

**Table 2:** Uni- and mult-ivariable regression analyses of the correlations of EEG and MRI abnormalities with demographic and clinical data in HIE patients undergoing hypothermia in 3 centers at high altitude in Multicenter Study

Variable	Background		Seizure time		Number of spikes/sharp waves		Number of delta brushes		Brain injury on MRI	
	Parameter estimate	*P* value	Parameter estimate	*P* value	Parameter estimate	*P* value	Parameter estimate	*P* value	Parameter estimate	*P* value
**Univariate**										
Center	0.32	0.005	73.37	0.40	20.52	0.002	-7.96	0.16	0.54	<0.001
Sex	0.05	0.75	70.32	0.58	1.14	0.92	-1.49	0.72	0.49	<0.001
Gestational age	-0.17	0.0003	-36.51	0.33	2.69	0.45	-6.33	<0.0001	0.014	0.46
Birthweight	-0.0004	0.039	0.15	0.23	-0.004	0.72	-0.02	0.0004	-0.0005	<0.001
Ethnics	0.15	0.10	-68.53	0.31	11.22	0.08	-1.07	0.64	0.40	<0.001
HIE degree	0.36	0.004	154.11	0.10	17.50	0.044	4.85	0.09	0.034	<0.001
Initiation age of hypothermia	0.03	0.003	-5.21	0.49	1.14	0.11	-0.01	0.98	0.034	<0.001
Treatment time	0.004	<0.0001	Time -5.92	0.40	Time -0.44 Time^2^ 0.01	<0.0001 <0.0001	Time -0.17 Time^2^ 0.001	0.002 0.040		
ScO_2_	0.002	0.42	0.87	0.63	-0.13	0.88	-0.072	0.58	0.0016	0.59
**Multivariate**										
Center	0.50	<0.0001			34.09	0.0003			0.04	<0.001
Sex									0.21	<0.001
Birthweight									-0.00046	<0.001
Ethnic							0.13	<0.0001	0.25	<0.001
HIE degree	0.22	0.050			6.18	0.013	6.18	0.013	0.30	<0.001
Initiation age of hypothermia									0.012	0.001
Treatment time	Time -0.001	<0.0001			Time -0.19 Time^2^ 0.01	<0.0001 <0.0001	0.14	<0.0001		
	Time^2^ 0.0001	0.02								

*Abbreviation*: *EEG* Electroencephalography, *MRI* Magnetic resonance imaging, *HIE* Hypoxic-ischemic encephalopathy

## Discussion

The incidence of HIE at high altitude is substantially higher compared to the developed countries; yet the proportion of patients undergoing TH (~20%) was substantially lower [1, 7, 9]. The clinical features are distinctively different in high-altitude regions. About one third of patients started TH later than 6 hours, and moderate and severe HIE accounted for 61%. Moreover, the 3 centers have very different features. Center 1’s Obstetrics Department is among the 10 best and largest centers in China; Center 2 is well known as ‘the Children’s Hospital’ in the province; Center 3 is a general hospital and has a relatively small NICU and Obstetrical Department. As a result, Center 1 had the largest number of HIE patients admitted and treated with TH, the mildest degree of HIE and the earliest age of TH, and better outcomes among the 3 centers. Furthermore, the ethnic composition is also different among centers. The majority of patients was Han in Center 1, representing the overall population composition in Qinghai; Hui in Center 2 since it is located in the area where Hui is the main habitants; all Tibetans in Center 3 as 90% of the population is Tibetans in Lhasa.

EEG also showed distinctive features in our cohort compared to previous reports [19, 25–27]. The abnormal discharges were mostly manifested as the micro-scale patterns, i.e., spikes/sharp waves and delta brushes. Seizures occurred only in 7-11%. Reports from low altitudes have universally reported seizures, with an incidence ranging 30~95% in patients undergoing TH [19, 25–27]. Altitude hypoxia may play a key role in the EEG alterations. The intrauterine environment already represents an extreme surrounding at sea level, which is exacerbated in pregnancies at high altitudes. Under altitude hypoxia, the utero-placental blood flow is lower, resulting in reduced oxygen uptake by the fetus [28]. This process can be worsened by preeclampsia and perinatal asphyxia [29]. Placental hypoxia and low birthweight at altitude have been reported [13, 14]. Speculatively, HIE patients born into hypoxic environments may have too limited capacity to manifest seizures which demand much energy and oxygen supply. A study using phosphorus-31 magnetic resonance spectroscopy has revealed that high-energy phosphates decrease by 33% and mitochondrial oxidative phosphorylation increases by 45% during neonatal seizures, indicating a depleted cerebral energy state [30]. When compared across 3 centers, EEG abnormalities of background and discharges were significantly greater, further supporting the hypothesis of altitude effect. After being adjusted by center, none of the EEG variables significantly correlated with demographic and clinical variables [19]. Paucity of data exists about pathophysiological and clinical meaning of spikes/sharp waves. One study examined the spikes/sharp waves and other EEG transients in neonates with neurologic risk conditions e.g., neonatal asphyxia [20]. A higher incidence of spikes/sharp waves was associated with worse neurological outcomes at 12 months of age. The number of delta brushes is usually used as a benchmark of neonatal brain immaturity and prognosis of adverse neurological outcomes [21, 31].

The EEG alternations and our hypothesis are supported by MRI findings. About 70~90% of patients across 3 centers had moderate and severe brain injuries after TH, which is substantially higher than previous reports (< 50%) [22, 25, 32]. The degree of brain injury on MRI correlated with the EEG parameters as well as demographic and clinical variables, Importantly, there was a linear and positive correlation between brain injury on MRI and initiation age of TH which ranged from 0.25~45 hours (1/3 of patients > 6 hours). At low altitudes, especially in advanced countries, newborns with untreated HIE after 6 hours are rare. The largest randomized trial involving 21 centers in the United State over 8 years provided an estimate of 24 hours during when TH remains effective.^8^ The therapeutic window may be narrower at high altitudes. Earlier initiation of TH, during transport for example [33], may be particularly beneficial for outborn patients at altitude.

The ethnic category as an independent adverse factor was a surprise. The opposite would be expected given the physiological and genetic adaptations of high altitude in Hui and Tibetan residents after living there for over hundreds to thousands of years. Tibetans are known to have the most optimal genetic adaptation to high altitudes following a process of natural selection through millennia [34, 35]. Han have only migrated there for about 70 years. Alternatively, this finding might be attributable to the socio-economic and cultural factors. The resources of health care remain limited in high-altitude regions worldwide. Many mothers lack the concept of adequate maternal care and regular checks during pregnancy. This problem was more serious in Tibetan, Hui, etc. than Han, similar to the findings in our previous study [15].

There were no serious complications related to TH in our cohort. Mortality was 5% during hospitalization and 9% after discharge before 1 year old. A recent report applying TH in low- and middle-income countries showed mortality of 36% during hospitalization and 11% by 18 months [36]. Mortality in our cohort is vastly different from this report, but close to other studies [3, 4]. Our results indicate that TH is safe and urgently needed. Considering the great severity of brain injury indicated by EEG and MRI, additional neuroprotective therapies may be necessary for these patients [37, 38], and hyperbaric oxygen makes sense [39].

The study has some limitations. 1) Clinical practice varied among the 3 centers despite uniform study protocol, which may have affected enrollment, treatment and outcomes. 2) Umbilical venous blood gas was lacking in patients transferred to communities. 3) We did not collect other risk factors related to altitudes such as maternal regular checks or rather not, placental abnormalities or fetal causes (e.g., intrauterine growth retardation). 4) We only examined the early effects of TH and mortality up to 1 year old. The assessments of early neurological and long-term neurodevelopmental outcomes were not made due largely to the lack of the personnel resources. Early identification of motor disabilities would be important for planning appropriate intervention in these infants [40]. The long-term neurodevelopmental outcome is particularly concerning since chronic hypoxia may adversely affect cognitive function in healthy residents at altitude [41].

## Conclusion

HIE at altitude is characterized by high incidence and severe brain injury as indicated by EEG and MRI. TH was effective, but only ~20%received TH with 1/3 initiated later than 6 hours. No major complications related to TH occurred.

### Supplementary Information

**Additional file 1.**

## Data Availability

The data used and analyzed for the study are available from the corresponding author on reasonable request.

## Abbreviations

ScO_2_: Cerebral oxygen saturation
HIE: Hypoxic-ischemic encephalopathy
TH: Therapeutic hypothermia
MRI: Magnetic resonance imaging
EEG: Electroencephalography
