# Supplementary material for: Characteristics of neonatal hypoxic-ischemic encephalopathy at high altitude and early results of therapeutic hypothermia
Source: BMC Pediatr. 2023 Dec 1;23:609. doi: 10.1186/s12887-023-04421-3 (PMC10691051; doi:10.1186/s12887-023-04421-3)
Supplement: Supplementary file 1 — Additional file 1. [file 12887_2023_4421_MOESM1_ESM.pdf]

Supplemental Table 1. Comparisons of EEG features among the 3 centers during 72-hour hypothermia and 24-hour rewarming in Multicenter Study

	Degree of background abnormalities			Seizure time (s)			Spike/sharp waves (times/h)			Delta brushes (times/h)			ScO2 (%)	
	Center 1	Center 2	Center 3	Center 1	Center 2	Center 3	Center 1	Center 2	Center 3	Center 1	Center 2	Center 3	Center 1	Center 2
**Time (hours)**														
0-4	1.0±0.7	2.0±1.0	1.8±1.0	0 (0- 0)	0 (0-0)	0 (0-0)	5.5 (0-86.8)	16.5 (0-270.5)	80 (9.8-266.3)	0 (0-20)	0 (0-0)	0.7 (0-31)	69.9±9.3	72.5±15.3
4-8	1.1±0.7	2.1±0.7	1.8±1.0	0 (0-800)	0 (0-0)	0 (0-0)	8 (0-128.8)	24.7 (0-148.5)	58.9 (4.3-375)	0 (0-131）	0 (0-10)	1.4 (0-32.8)	70.2±7.3	63.5±7.0
8-12	1.1±0.7	2.3±0.8	1.9±1.1	0 (0-0)	0 (0-0)	0 (0-0)	5 (0-113.5)	41.9 (2-155)	56.8 (0-255)	0 (0-98)	0 (0-2.8)	1.2 (0-62)	70.9±9.8	62.5±7.2
12-16	1.1±0.8	2.0±0.9	1.9±1.2	0 (0-0)	0 (0-0)	0 (0-0)	5.8 (0-158.3)	18.5 (0-119.3)	68.4 (0-410)	0 (0-69)	0 (0-1.8)	0.8 (0-48.5)	69.6±9.8	67.8±4.6
16-20	1.1±0.7	1.8±0.8	2.0±1.1	0 (0-0)	0 (0-685)	0 (0-0)	11.8 (0-116)	14.8 (0-160)	50 (0-345)	0 (0-60.5)	0 (0-8)	2.3 (0-32)	70.5±10.9	63.5±5.7
20-24	1.1±0.7	2.0±0.9	1.9±1.1	0 (0-0)	0 (0-0)	0 (0-0)	10.3 (0-124)	18.3 (0-75)	30.5 (3.5-292.5)	0 (0-128)	0 (0-0)	1.5 (0-50)	70.8±9.5	65.8±5.0
24-28	1.0±0.8	1.8±0.9	2.4±0.7	0 (0-0)	0 (0-0)	0 (0-0)	5.5 (0-124.3)	21.5 (0-308.8)	98.1 (1.8-297.5)	0 (0-79.8)	0 (0-2)	0.9 (0-64)	70.4±9.8	60.6±9.2
28-32	0.9±0.8	2.0±0.8	2.5±0.5	0 (0-0)	0 (0-580)	0 (0-0)	7 (0-117)	32 (0-550)	64.9 (0-222.5)	0 (0-150)	0 (0-2.3)	1 (0-42.5)	71.6±10.6	60±9.3
32-36	0.9±0.8	1.9±1.0	2.3±0.7	0 (0-0)	0 (0-0)	0 (0-0)	8 (0-100)	17.5 (0-525)	66.6 (6-322.5)	0 (0-219)	0 (0-0)	1.5 (0-90)	71.4±10.0	59.6±8.8
36-40	0.9±0.7	1.6±1.2	2.4±0.5	0 (0-0)	0 (0-0)	0 (0-0)	8.3 (0-100)	18.7 (0-475)	80.8 (1.3-222)	0 (0-57.5)	0 (0-0.5)	1.3 (0-56.3)	70.5±10.1	61.4±13.0
40-44	0.9±0.7	1.3±1.2	1.9±1.1	0 (0-0)	0 (0-0)	0 (0-0)	8.5 (0-140)	23.8 (2.3-440)	81.6 (0-277.5)	0 (0-33)	0 (0-1.8)	0 (0-15)	70.6±10.3	56.8±5.5
44-48	1.0±0.7	1.6±1.2	1.8±1.0	0 (0-0)	0 (0-0)	0 (0-0)	7 (0-123.3)	17.5 (0-526.7)	71.3 (21-181.3)	0 (0-224)	0 (0-2.5)	0 (0-4.5)	72.4±11.0	53.4±8.3
48-52	1.3±0.7	1.9±0.9	2.0±1.0	0 (0-0)	0 (0-0)	0 (0-840)	9.5 (0-111.8)	14.1 (0-577.5)	60 (7-197.5)	0 (0-45)	0 (0-0)	0 (0-7.5)	72.2±11.8	58±14.8
52-56	1.3±0.7	1.8±1.2	1.9±1.0	0 (0-0)	0 (0-0)	0 (0-1680)	10.5 (0-118.3)	21.6 (0-505)	63.8 (4-133.3)	0 (0-137.8)	0 (0-3.3)	2.3 (0-12.8)	70.9±10.9	58.6±10.3
56-60	1.3±0.7	1.9±1.3	1.9±1.0	0 (0-0)	0 (0-0)	0 (0-510)	16.5 (0-96.3)	21.4 (0-525)	86.3 (7.8-211.3)	0 (0-73.5)	0 (0-3.3)	0.9 (0-20)	72.9±10.9	52±12.9
60-64	1.4±0.7	2.2±1.2	1.8±1.0	0 (0-0)	0 (0-0)	0 (0-0)	12 (0-107.3)	26.8 (0-305)	51.3 (5.5-262.5)	0 (0-77.3)	0 (0-3.3)	0.5 (0-6.5)	73.2±9.8	61.8±11.5
64-68	1.3±0.7	2.1±1.0	1.8±1.0	0 (0-230)	0 (0-0)	0 (0-0)	10.5 (0-68.8)	15 (0-114.3)	104.9 (6.5-317.5)	0 (0-20)	0 (0-8.8)	0.3 (0-12.3)	72.1±11.2	55.6±7.5
68-72	1.4±0.8	2.3±0.8	1.8±1.0	0 (0-0)	0 (0-0)	0 (0-0)	12.1 (0-79.5)	17.4 (0-225)	105 (7-500)	0 (0-58.8)	0 (0-6)	0 (0-17.5)	72.9±9.3	61.8±14.9
72-76	1.4±0.7	2.1±0.9	1.9±0.9	0 (0-120)	0 (0-0)	0 (0-0)	18.5 (0-128.5)	25 (0-168.8)	97.3 (10-480)	0 (0-90)	0 (0-4.5)	2.2 (0-5.3)	72.6±10.7	56.5±23.3
76-80	1.3±0.6	2.0±0.8	2.0±0.9	0 (0-168)	0 (0-0)	0 (0-0)	29 (3.3-129.3)	50.6 (0-238.8)	103.1 (16.5-610)	0 (0-87.3)	0 (0-12)	0 (0-6.8)	74.2±11.2	66.5±26.2
80-84	1.3±0.7	2.6±0.5	1.9±0.9	0 (0-0)	0 (0-0)	0 (0-0)	40.1 (1.8-132)	73 (0-237.5)	99.5 (16-635)	0 (0-117.5)	0 (0-1)	0 (0-39.5)	72.2±11.6	70±28.3
84-88	1.4±0.6	2.4±0.7	2.0±1.1	0 (0-80)	0 (0-0)	0 (0-0)	41.3 (5.7-163)	81.8 (0-212.3)	80.5 (11.3-645)	0 (0-120.8)	0 (0-4.3)	0 (0-27.5)	77.9±7.7	84±10.0
88-92	1.4±0.7	2.5±0.5	1.9±1.1	0 (0-0)	0 (0-0)	0 (0-0)	31.5 (7.3-203)	98.0 (0-276)	73.3 (9.3-563.3)	0 (0-88.5)	0 (0-5.3)	1.8 (0-46.8)	77.9±10.2	80.0±6.5
92-96	1.5±0.6	2.4±0.5	1.9±1.1	0 (0-0)	0 (0-0)	0 (0-0)	47.3 (8.7-167.5)	76.8 (0-465)	95 (4-208.7)	0 (0-14.3)	0 (0-7)	3.8 (0-36.3)	72±13	68.5±9.2
**Statistical analysis**														
**Center**														
Parameter estimate		0.50			-19.50			117.3			-84.0			2.73
P value		<0.001			0.59			<0.001			0.65			0.01
**Time**														
Parameter estimate		-0.002			-0.56			-1.20			0.175			0.142
P value		<0.001			0.32			<0.001			<0.001			<0.001
**Time^2**														
Parameter estimate		0.0001						0.011						-0.001
P value		0.01						<0.001						<0.001

Abbreviation: EEG = Electroencephalography

Center 1 = Qinghai Red Cross Hospital; Center 2 = Qinghai Women and Children's Hospital; Center 3 = Lhasa People's Hospital.
